# Supplementary material for: Weighted Gene Co-Expression Network Analysis Identifies a Functional Guild and Metabolite Cluster Mediating the Relationship between Mucosal Inflammation and Adherence to the Mediterranean Diet in Ulcerative Colitis
Source: Int J Mol Sci. 2023 Apr 15;24(8):7323. doi: 10.3390/ijms24087323 (PMC10138710; doi:10.3390/ijms24087323)
Supplement: Supplementary file 1 [file ijms-24-07323-s001.zip › ijms-2299028-supplementary.pdf]

Title: Identification of a functional guild of microbial taxa and metabolite cluster associated with the Mediterranean diet and mucosal inflammation in Ulcerative Colitis through weighted gene co-expression network analysis (WCGNA)

Authors:

*J. Strauss[1], *N. Haskey[2], H. Ramay[3], T. Ghosh[4], L.M. Taylor[1], M. Yousuf[1], C. Ohland[5], K.D. McCoy[5], J.M. Ingram[1], S. Ghosh[4], R. Panaccione[1], and M. Raman[1]

Affiliations:

1. Department of Medicine, Cumming School of Medicine, University of Calgary, 2500 University Drive NW, Calgary, Alberta, Canada T2N 1N4
2. Department of Biology, Irving K Barber Faculty of Science, University of British Columbia – Okanagan, 3137 University Way, Kelowna, British Columbia, Canada V1V 1V7
3. International Microbiome Centre, HRIC 4AA08 Foothills Campus, University of Calgary, Calgary, Alberta, Canada T2N 1N4
4. Department of Computational Biology, Indraprastha Institute of Information Technology Delhi (IIIT-Delhi), Okhla Industrial Estate, Phase III, New Delhi, India,110020
5. APC Microbiome Ireland, College of Medicine and Health, University College Cork, College Road, National University of Ireland, Cork, Ireland T12 K8AF

Online Supplementary Material

Table of Contents

1. Extended Supplementary Methods ... *3*

1.1. Details of DNA isolation of fecal samples ... 3

1.2. Metabolomic Analyses ... 3

1.2.1. Mass to Charge Ratios of Bile Acids ... 3

1.2.2. Preparation of the Calibration Solution for Bile Acids ... 3

2. Protocol Deviations ... *3*

3. Supplementary Tables ... *4*

Table S1. Demographic description of all the participants recruited to the open-label study (n = 40). ... 4

Table S2. Medication of all the participants recruited to the open-label study (n = 40). ... 5

Table S3. Diet characteristics of all the participants recruited to the open-label study (n = 40). ... 5

Table S4. Fecal short-chain fatty acids of all the participants recruited to the open-label study (n = 32)*. ... 6

Table S5. Bile acids of all the participants recruited to the open-label study (n = 33)*. ... 6

Table S6. The association between diet (adjusted and unadjusted)† and fecal calprotectin levels at baseline, 8 weeks and the change from baseline to 8 weeks (n = 29)*. ... 6

Table S7. No significant association between the change in fecal calprotectin from baseline to 8 weeks and the MD intervention were observed. ... 7

Table S8. The association between diet (adjusted and unadjusted)[a,†] and fecal calprotectin at baseline, 8 weeks and the change from baseline to 8 weeks (n = 29)*. (A) The baseline diet (both adjusted and unadjusted) are associated with the change in fecal calprotectin from baseline to 8 weeks. (B) The change in diet over time (adjusted macronutrients + micronutrients and unadjusted macronutrients + micronutrients) is significantly associated with the baseline fecal calprotectin, fecal calprotectin at week 8 and the change in fecal calprotectin from baseline to 8 weeks. ... 7

Table S9. Actual correlational values and p-values for each module in the WGCNA. ... 8

4. Supplementary Figures ... *9*

Figure S1. Consort flow diagram. ... 9

Figure S2. Plot of the Spearman rho correlation results shows the change in Mediterranean diet score and change in fecal calprotectin. The blue line is the best fit through the data. ... 10

Figure S3. Spearman rho correlation plot showing the relationship between the change in species abundance with the fecal calprotectin change versus the change in species abundance with the Mediterranean diet score change. ... 11

Figure S4. Overview of the weighted gene co-expression analyses used to identify modules ... 12

Figure S5. The weighted gene co-expression analyses identified 10 modules containing 362 features that associate with FCP and Mediterranean diet score (MDS) ... 12

References ... *13*

Online Supplementary Material

1. Extended Supplementary Methods

1.1. Details of DNA isolation of fecal samples

Fecal samples were collected at baseline and week 8. Samples were immediately frozen at −20 °C, transferred to the IMC, and stored at −80 °C until DNA extraction. Whole genomic DNA was extracted using the PowerSoil Pro DNA kit (Qiagen) according to the manufacturer's instructions. The 16S rRNA gene V4 variable region was amplified using PCR primers with internal barcodes in a 25 cycle PCR using the KAPA HiFi HotStart master mix (Roche Sequencing). The PCR products were then purified using NucleoMag NGS Clean-up and Size Select (Macherey-Nagel) and concentrations normalized using SequalPrep Normalization Plate (Invitrogen). Amplicons were pooled and concentration and quality were determined using the Qubit HS DNA kit (Invitrogen) and the Tapestation D1000 assay (Agilent Technologies), respectively. Amplicon sequencing was done on a MiSeq Benchtop DNA sequencer (Illumina) using a V2-500 cycle kit (Illumina Inc). The pooled library was then denatured and prepared for loading on an Illumina MiSeq cartridge with a 5% PhiX Control.

1.2. Metabolomic Analyses

1.2.1. Mass to Charge Ratios of Bile Acids

CA, *m*/*z* 426.3 → *m*/*z* 355.3 (20), *m*/*z* 373.3 (14); CDCA/DCA/HDCA/UDCA, *m*/*z* 410.3 → *m*/*z* 357.3 (14), *m*/*z* 339.3 (15); CDCA-Me, *m*/*z* 508.4 → *m*/*z* 371.3 (17), *m*/*z* 339.3 (22); GCA, *m*/*z* 483.3 → *m*/*z* 412.4 (20), *m*/*z* 430.4 (17); GCDCA, *m*/*z* 467.4 → *m*/*z* 414.3 (19), *m*/*z* 432.4 (13); LCA, *m*/*z* 394.3 → *m*/*z* 359.4 (12), *m*/*z* 135.4 (35); TCA, *m*/*z* 533.3 → *m*/*z* 462.3 (22), *m*/*z* 337.3 (28); TCDCA,/TDCA *m*/*z* 517.3 → *m*/*z* 464.4 (19), *m*/*z* 482.4 (13); TLCA, *m*/*z* 501.3 → *m*/*z* 466.4 (16), *m*/*z* 341.3 (24).

Abbreviations: CA, cholic acid; CDCA, chenodeoxycholic acid; CDCA-Me, chenodeoxycholic acid diacetate methyl ester; DCA, deoxycholic acid; GCA, glycocholic acid; GCDCA, glycochenodeoxycholic acid; HDCA, hyodeoxycholic acid; LCA, lithocholic acid; TCA, taurocholic acid; TCDCA, taurochenodeoxycholic acid; TDCA, taurodeoxycholic acid; TLCA, taurolithocholic acid; UDCA, ursodeoxycholic acid.

1.2.2. Preparation of the Calibration Solution for Bile Acids

A freshly prepared working solution containing CA, CDCA, DCA, HDCA, LCA and UDCA at 200 μM (final concentration), GCA, GCDCA, TCA, TCDCA, TDCA at 50 μM (final concentration), TLCA at 30 μM (final concentration) and CDCA-Me at 0.5 μM (final concentration) in MeOH, was further diluted (1:2) in MeOH to prepare 12 calibration solutions.

2. Protocol Deviations

In March 2020, the University of Calgary curtailed all campus research which impacted recruitment. Due to challenges with sample size attainment, we expanded the inclusion criteria to include patients in remission (defined by partial Mayo score < 2) and to patients with a moderate disease flare (defined by partial Mayo score >2). Endoscopic assessments could not be consistently completed due to the high demand for service in the endoscopic unit.

3. Supplementary Tables

Table S1. Demographic description of all the participants recruited to the open-label study (n = 40).

	**Habitual Diet (*n* = 18) median (IQR)**			**Intervention (*n* = 22) median (IQR)**			**Between Groups**	
	**Baseline**	**Week 8**	**P-value[a]**	**Baseline**	**Week 8**	**P-value[a]**	**Baseline[b]**	**Week 8[b]**
Patient characteristics								
Male, *n* (%)	11 (61)	-	-	10 (45)	-	-	0.36	-
Age (years)	32 (27–45)	-	-	38 (34-47)	-	-	0.21	-
Total Mayo Score	6.0 (4.5–7.0) (*n* = 13)	2.0 (0.25–3.0) (*n* = 8)	**0.02**	6.0 (1.0–7.5) (*n* = 17)	0.50 (0–1.8) (*n* = 12)	**0.008**	0.41	0.20
Body mass index (Kg/m^2)	25 (24–30) (*n* = 16)	26 (25–32) (*n* = 14)	**0.03**	26 (23–28) (*n* = 21)	25 (21–27) (*n* = 15)	0.16	0.41	0.06
Partial Mayo Score	3.5 (1.0–5.0)	1.0 (0–1.8)	**0.007**	2.0 (0–5.0) (*n* = 21)	0 (0–1.0) (*n* = 17)	**0.003**	0.36	0.22
*Partial Mayo Score by disease activity^*								
Remission, n (%)	6 (33%)	13 (81%)	**0.04**	9 (43%)	15 (88%)	**0.02**	0.70	0.93
Mild, n (%)	7 (39%)	2 (13%)	0.49	5 (24%)	1 (6%)	0.69	0.58	0.85
Moderate, n (%)	3 (17%)	1 (6%)	0.79	4 (19%)	0	n/a	0.95	n/a
Severe, n (%)	2 (11%)	0	n/a	3 (14%)	1 (6%)	0.83	0.92	n/a
Fecal Calprotectin								
Fecal Calprotectin (mcg/g)	235 (73–3945) (*n* = 17)	271 (73–2435) (*n* = 16)	0.68	73 (30–989) (*n* = 22)	172 (37–844) (*n* = 18)	0.72	0.08	0.23
FCP < 100, remission n (%)[†^]	9 (53%)	6 (38%)	0.57	12 (55%)	9 (50%)	0.82	0.93	0.67
FCP 100–250, grey zone n (%)[†^]	2 (12%)	2 (12%)	1.00	2 (9%)	3 (17%)	0.80	0.92	0.88
FCP > 250, active n (%)[†^]	6 (35%)	8 (50%)	0.58	8 (36%)	6 (33%)	0.93	0.97	0.52

Abbreviations: IQR, interquartile range. Statistics P <0.05, [a]Wilcoxon-signed rank test, [b]Mann-Whitney test, n/a, insufficient counts to perform statistic, ^test of proportions, [†]based on the International Organization for the Study of IBD guidelines [1].

Table S2. Medication of all the participants recruited to the open-label study (n = 40).

Medications	Habitual Diet (n = 18) median (IQR)			Intervention (n = 22) median (IQR)		
	Baseline	Week 8	P-value[a]	Baseline	Week 8	P-value[a]
Corticosteroid *n* (%)	5 (28%)	5 (28%)	NS	8 (36%)	8 (36%)	NS
5-ASA monotherapy *n* (%)	14 (78%)	14 (78%)	NS	15 (68%)	15 (68%)	NS
Anti-TNF *n* (%)	4 (22%)	4 (22%)	NS	6 (27%)	6 (27%)	NS
Immunomodulator *n* (%)	3 (14%)	3 (14%)	NS	3 (14%)	3 (14%)	NS
Probiotics *n* (%)	3 (17%)	3 (17%)	NS	6 (27%)	6 (27%)	NS
Antibiotics *n* (%)	2 (11%)	2 (11%)	NS	4 (18%)	4 (18%)	NS

Abbreviations: 5-ASA:5-aminosalicylic acids; Anti-TNF: anti-tumour necrosis factor. IQR, interquartile range; NS, not significant. Statistics P <0.05.

Table S3. Diet characteristics of all the participants recruited to the open-label study (n = 40).

Diet	Habitual Diet (n = 18) median (IQR)			Intervention (n = 22) median (IQR)			Between Groups	
	Baseline	Week 8	P-value[a]	Baseline	Week 8	P-value[a]	Baseline[b]	Week 8[b]
Sulfur (g/day)*	159 (44) (*n* = 12)	151 (83) (*n* = 12)	0.66[c]	178 (72) (*n* = 17)	134 (56) (*n* = 17)	**0.005**[c]	0.32[d]	0.53[d]
Sulfur/1000 kcal**	69 (58–90) (*n* = 12)	69 (45–93) (*n* = 12)	0.73	71 (58–107) (*n* = 17)	80 (64–90) (*n* = 17)	0.89	0.53	0.37
Mediterranean diet score (MDS)***	5 (4.0–6.0)	5 (4.0–6.0) (*n* = 11)	0.87	5 (4.0–6.0)	5 (3.3–6.8) (*n* = 16)	0.65	0.57	0.69

Abbreviations: IQR, interquartile range. Statistics P < 0.05, *Absolute grams of sulfur in the diet, mean (standard deviation), **Sulfur normalized to 1000 kcal, ***Highest achievable score is 13, [a]Wilcoxon-signed rank test, [b]Mann-Whitney test, [c]Paired *t*-test, [d]Unpaired t-test.

Table S4. Fecal short-chain fatty acids of all the participants recruited to the open-label study (n = 32)*.

Short Chain Fatty Acids (μM)	Habitual Diet (*n* = 15) median (IQR)			Intervention (*n* = 17) median (IQR)			Between Groups	
	Baseline	Week 8	P-value[a]	Baseline	Week 8	P-value[a]	Baseline[b]	Week 8[b]
Acetate	19815 (16332–27309)	15362 (7187–26903)	0.42	17055 (10768–26819)	18055 (1403–22369)	>0.99	0.41	0.77
Propionate	5976 (5189–8981)	4479 (2325–6961)	0.28	4866 (2862–8371)	5288 (3479–6299)	>0.99	0.25	0.71
Butyrate	7008 (4387–9895)	5302 (2177–7024)	0.28	5520 (3362–7354)	5649 (4539–6167)	0.77	0.16	0.50
Iso-Butyrate	469 (265–569)	361 (246–594)	>0.99	472 (360–691)	645 (437–836)	0.58	0.35	0.09
Valerate	439 (105–722)	360 (58–677)	0.77	598 (423–944)	704 (441–958)	0.71	0.23	**0.05**
Iso-Valerate	362 (220–1669)	318 (211–548)	0.75	395 (248–740)	562 (343–803)	0.26	0.39	0.07

Abbreviations: IQR, interquartile range. Statistics p <0.05, [a]Wilcoxon-signed rank test, [b]Mann-Whitney test, *data only available for 32 participants.

Table S5. Bile acids of all the participants recruited to the open-label study (n = 33)*.

Bile Acids (μM)	Habitual (*n* = 16) median (IQR)			Intervention (*n* = 17) median (IQR)			Between Groups	
	Baseline	Week 8	P-value[a]	Baseline	Week 8	P-value[a]	Baseline[b]	Week 8[b]
Cholic Acid	7.7 (2.2–70)	21.0 (1.8–348)	0.63	1.7 (0.75–6.3)	2.9 (0.60–29)	0.44	0.08	0.17
Chenodeoxycholic acid	5.8 (2.0–21)	3.1 (1.1–68)	0.35	1.5 (0.93–3.7)	1.9 (0.60–17)	0.80	0.03	0.54
Deoxycholic acid	164 (99–309)	234 (125–352)	0.30	140 (27–347)	135 (106–302)	0.98	0.74	0.25
Glycochenodeoxy cholic acid	0.40 (0.20–0.60)	0.40 (0.23–0.70)	>0.99	0.10 (0.10–0.30)	0.20 (0.10–0.30)	0.59	**0.005**	**0.02**

Abbreviations: IQR, interquartile range. Statistics p <0.05, [a]Wilcoxon-signed rank test, [b]Mann-Whitney test, *data only available for 33 participants.

Table S6. The association between diet (adjusted and unadjusted)† and fecal calprotectin levels at baseline, 8 weeks and the change from baseline to 8 weeks (n = 29)*.

Terms	PERMANOVA Intervention Status	
	R-Squared	P-Value
Baseline Diet (Unadjusted)	0.047	0.64
Baseline Diet (Adjusted)	0.056	0.29
8 Week Diet (Unadjusted)	0.047	0.62
8 Week Diet (Adjusted)	0.053	0.43
Diet Change (Unadjusted)	0.049	0.57
Diet Change (Adjusted)	0.056	0.35

*29 subjects with matching fecal calprotectin and diet data; unadjusted refers to absolute values, adjusted refers to normalized value to 1000 kcal. †*Further details about which specific nutrients included in the analyses can be found in the*

supplementary info: UC_Total_Macro_Micro.txt, UC_Freq_Food_Groups.txt, UC_Adj_Macro_Micro.txt stored in Strauss & Haskey et al. (2023), "Weighted gene co-expression network analysis identifies a functional guild and metabolite cluster mediating the relationship between mucosal inflammation and adherence to the Mediterranean diet in Ulcerative Colitis", Mendeley Data, V1, doi: 10.17632/rt3682s6kn.1.

Table S7. No significant association between the change in fecal calprotectin from baseline to 8 weeks and the MD intervention were observed.

Change Status	Intervention	Controls
FCP increased over time	6	6
FCP decreased over time	7	9

Fisher's exact test P-value = 1, estimate 1.27, Abbreviations: FCP, fecal calprotectin.

Table S8. The association between diet (adjusted and unadjusted)[a,†] and fecal calprotectin at baseline, 8 weeks and the change from baseline to 8 weeks (n = 29)*. (A) The baseline diet (both adjusted and unadjusted) are associated with the change in fecal calprotectin from baseline to 8 weeks. (B) The change in diet over time (adjusted macronutrients + micronutrients and unadjusted macronutrients + micronutrients) is significantly associated with the baseline fecal calprotectin, fecal calprotectin at week 8 and the change in fecal calprotectin from baseline to 8 weeks.

A

Terms	PERMANOVA (Baseline Diet - UnAdjusted)	
	**R-Squared**	**P-Value**
FCP (Baseline)	**0.056**	**0.036***
FCP (8 week)	0.045	0.14
FCP Change Across Time	**0.065**	**0.006****

Terms	PERMANOVA (Baseline Diet - Adjusted)	
	**R-Squared**	**P-Value**
FCP (Baseline)	0.049	0.11
FCP (8 week)	0.039	0.44
FCP Change Across Time	**0.07**	**0.008**

B

Terms	PERMANOVA (Diet Change Across Time - UnAdjusted)[b]	
	**R-Squared**	**P-Value**
FCP (Baseline)	**0.091**	**0.002****
FCP (8 week)	**0.071**	**0.048***
FCP Change Across Time	**0.081**	**0.022***

Terms	PERMANOVA (Diet Change Across Time - Adjusted)[c]	
	**R-Squared**	**P-Value**
FCP (Baseline)	**0.093**	**0.003****
FCP (8 week)	**0.071**	**0.06 @**
FCP Change Across Time	**0.085**	**0.012***

*29 subjects with matching fecal calprotectin and diet data. [a]unadjusted refers to absolute values, adjusted refers to normalized value to 1000 kcal. [b]unadjusted macronutrients + micronutrients and [c]adjusted macronutrients + micronutrients, *†Further details about which specific nutrients included in the analyses can be found in the supplementary info: UC_Total_Macro_Micro.txt, UC_Freq_Food_Groups.txt, UC_Adj_Macro_Micro.txt stored in Strauss & Haskey et al. (2023), "Weighted*

gene co-expression network analysis identifies a functional guild and metabolite cluster mediating the relationship between mucosal inflammation and adherence to the Mediterranean diet in Ulcerative Colitis", Mendeley Data, V1, doi: 10.17632/rt3682s6kn.1.

Table S9. Actual correlational values and p-values for each module in the WGCNA.

	FCP_association_r	FCP_association_p	MDS_association_r	MDS_association_p
Black	0.06316969	0.61713975	−0.0436935	0.74690365
Blue	0.29414746	0.01739009	−0.1667427	0.21508996
Brown	−0.0332329	0.79270042	−0.2311042	0.08369403
Green	−0.1899466	0.1296412	0.42564846	0.00096374
Grey	0.03133828	0.80427694	0.03828514	0.77737124
Magenta	0.12162073	0.33449431	−0.0583042	0.66661261
Pink	0.13274502	0.29182229	0.12440968	0.35650129
Red	0.09725738	0.44086731	−0.0166886	0.90193805
Turquoise	0.01301676	0.91803242	0.14932531	0.26758795
Yellow	−0.030829	0.80739609	−0.2955966	0.02558804

Abbreviations: FCP, fecal calprotectin; MDS, Mediterranean diet score.

4. Supplementary Figures

CONSORT
TRANSPARENT REPORTING of TRIALS

CONSORT 2010 Flow Diagram for UC Study

Enrollment

Assessed for eligibility (n=167)

Excluded (n=113)
- Not meeting inclusion criteria (n= 3)
- Declined to participate (n=107)
- Other reasons (n=11)

Randomized (n=46)

Allocation

Allocated to intervention (n=26)
- Received allocated intervention (n=25)
- Did not receive allocated intervention (study failure) (n= 1)

Allocated to control (n=20)
- Received normal standard of care (n=20)
- Did not receive allocated intervention (give reasons) (n= 0)

Follow-Up

Lost to follow-up (Covid pandemic, stopped responding) (n=3)

Discontinued intervention (give reasons) (n=0)

Lost to follow-up (Covid pandemic) (n=2)

Discontinued Study (give reasons) (n=0)

Analysis

Analysed (n=22)
- Excluded from analysis (n=0)

Analysed (n=18)
- Excluded from analysis (n=0)

Figure S1. Consort flow diagram.

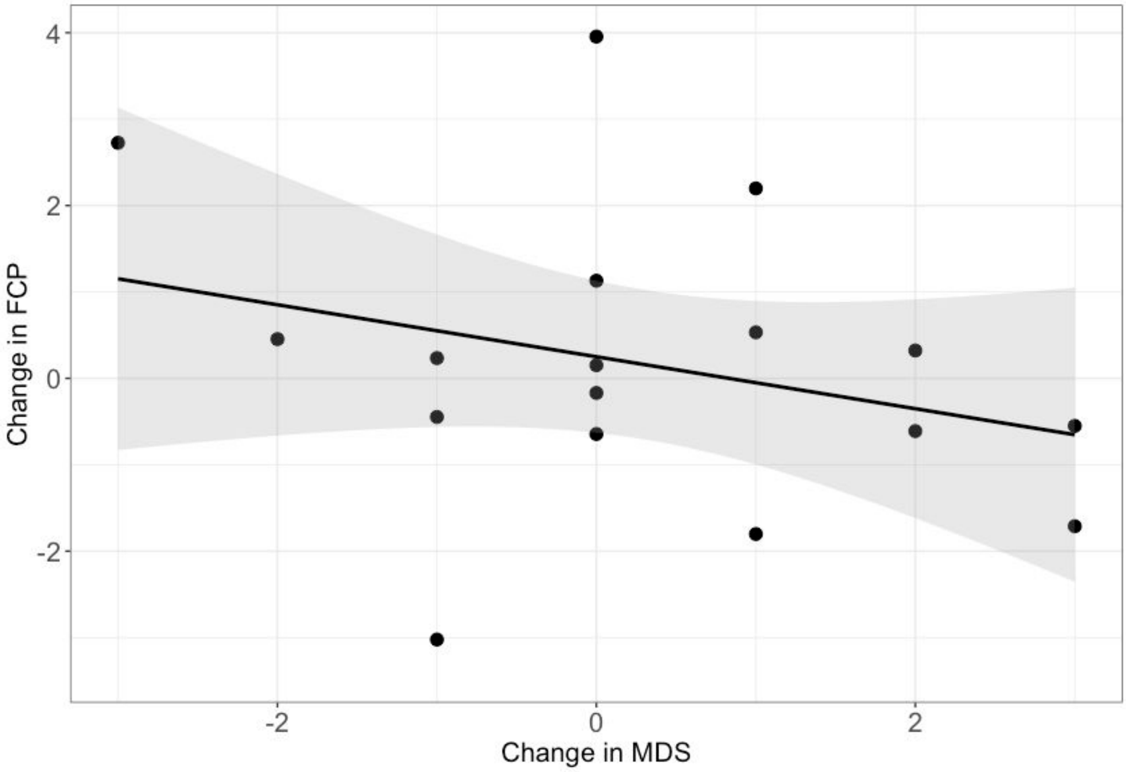

Figure S2. Plot of the Spearman rho correlation results shows the change in Mediterranean diet score and change in fecal calprotectin. The blue line is the best fit through the data.

A positive change in the Mediterranean diet score is associated with a negative change in fecal calprotectin (Spearman Rho = −0.27 and P = 0.29).

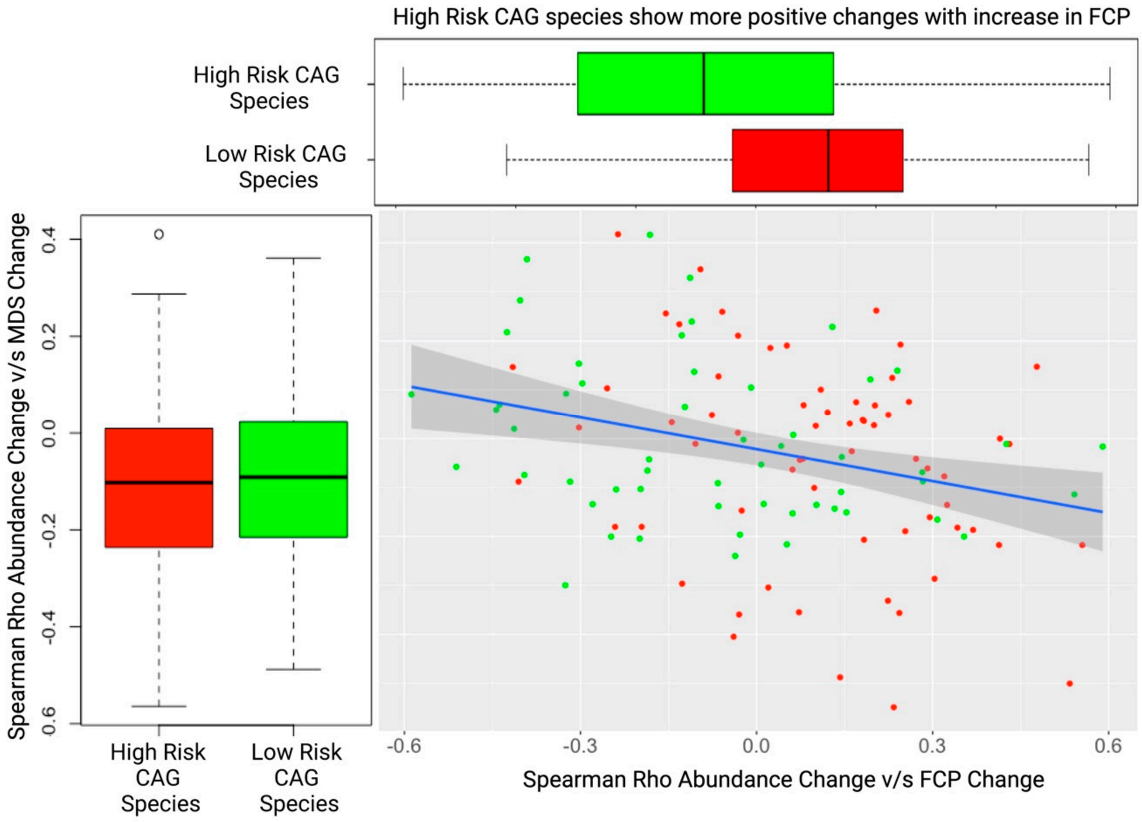

Figure S3. Spearman rho correlation plot showing the relationship between the change in species abundance with the fecal calprotectin change versus the change in species abundance with the Mediterranean diet score change.

The more positively associated the change in species abundance with the fecal calprotectin change, the more negative the association with change in species abundance with the Mediterranean diet score change. The blue line is the best fit through the data (Spearman's Rho is −0.29 and p-value is 0.0012).

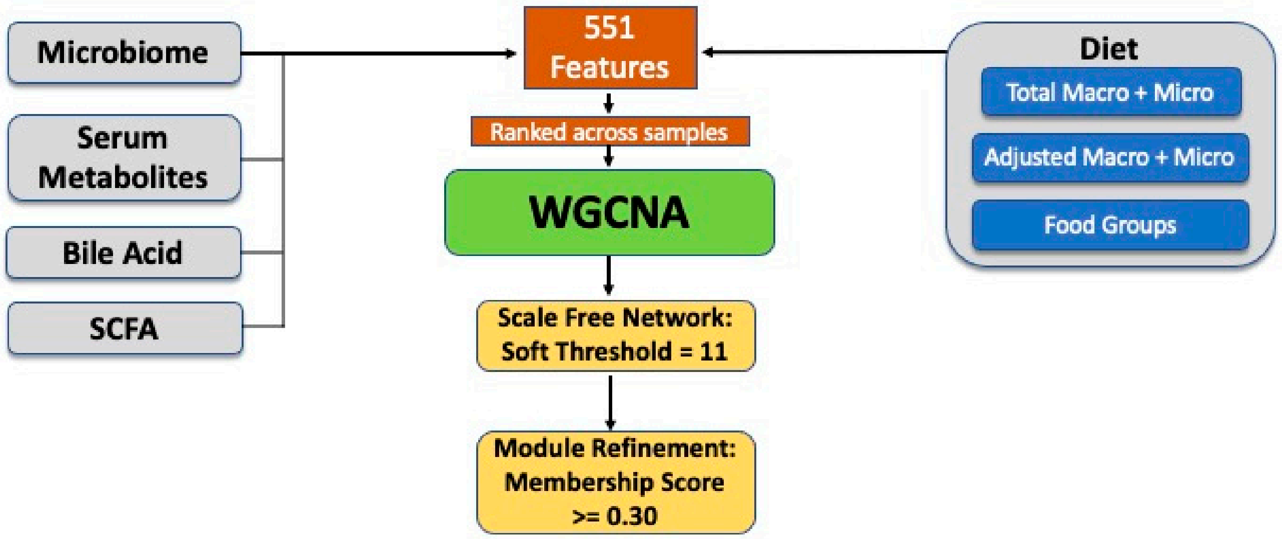

Figure S4. Overview of the weighted gene co-expression analyses used to identify modules.

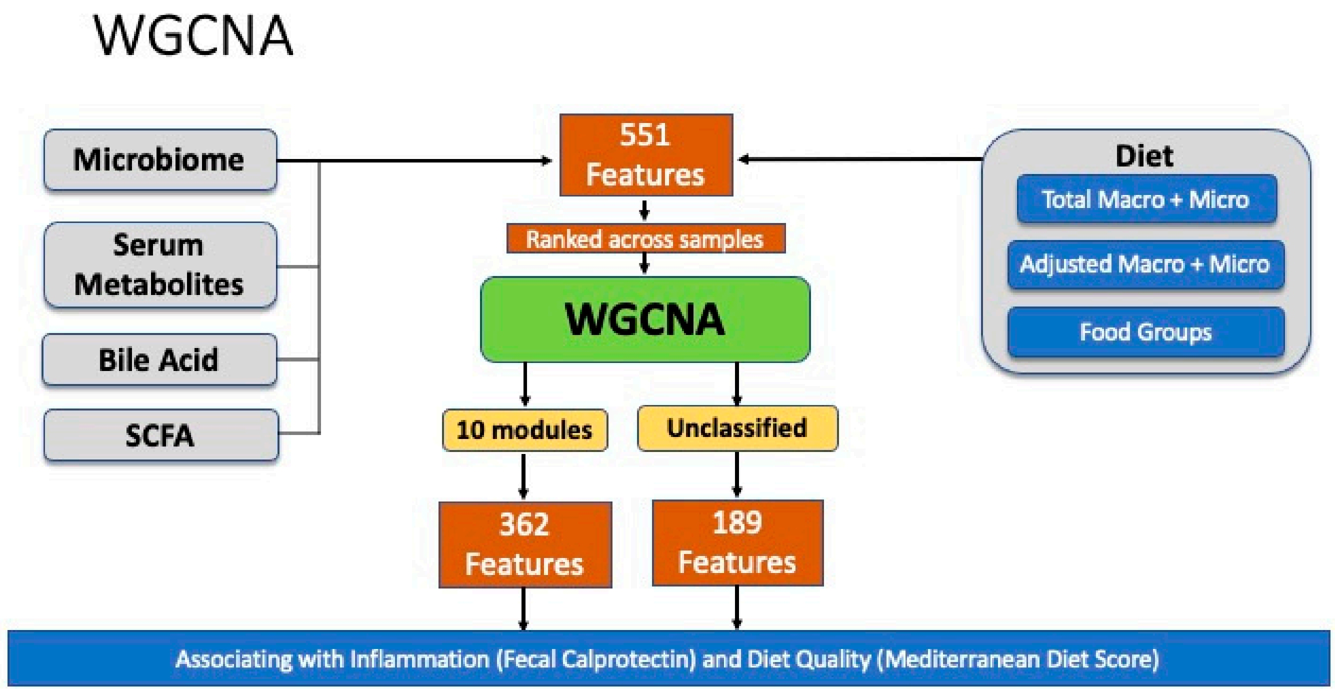

Figure S5. The weighted gene co-expression analyses identified 10 modules containing 362 features that associate with FCP and Mediterranean diet score (MDS).

Online Supplementary Material

References

1. Turner, D.; Ricciuto, A.; Lewis, A.; D’Amico, F.; Dhaliwal, J.; Griffiths, A. M.; Bettenworth, D.; Sandborn, W. J.; Sands, B. E.; Reinisch, W.; et al. STRIDE-II: An Update on the Selecting Therapeutic Targets in Inflammatory Bowel Disease (STRIDE) Initiative of the International Organization for the Study of IBD (IOIBD): Determining Therapeutic Goals for Treat-to-Target Strategies in IBD. *Gastroenterology*, **2021**, *160* (5). https://doi.org/10.1053/j.gastro.2020.12.031.
